# Feasibility of laparoscopic gastrectomy for patients with Siewert-type II/III adenocarcinoma of the esophagogastric junction: A propensity score matching analysis

**DOI:** 10.1371/journal.pone.0203125

**Published:** 2018-09-26

**Authors:** Yinan Shi, Linjie Li, Huashi Xiao, Shanshan Guo, Guiping Wang, Kai Tao, Jianhong Dong, Liang Zong

**Affiliations:** 1 Department of minimal invasive gastrointestinal surgery, Shanxi Cancer Hospital, affiliated to Shanxi Medical University, Taiyuan, Shanxi, PR. China; 2 Department of Gastrointestinal Surgery, Clinical Medical School of Yangzhou university, Northern Jiangsu People’s Hospital, Yangzhou, Jiangsu, PR. China; 3 Clinical Medical College, Dalian Medical University, Liaoning, PR. China; National Cancer Center, JAPAN

## Abstract

**Background/Aim:**

The feasibility of using laparoscopic gastrectomy for the treatment of Siewert-type II/III adenocarcinoma of the esophagogastric junction (AEG) has not been addressed. This study aimed to comparatively evaluate the short- and long-term effects on laparoscopic versus open surgery using (propensity score matching) PSM for Siewert-type II/III AEG.

**Methods:**

We retrospectively collected data from the patients with Siewert-type II/III AEG who were treated in our cancer center between January 2013 and December 2015. Patients undergoing laparoscopic gastrectomy and open gastrectomy were matched via PSM. The cumulative 2-year Overall survival (OS) rate of patients in the two cohorts was estimated by Kaplan-Meier plots. Multi-variable analysis using a Cox regression model was conducted to identify independent risk factors.

**Results:**

A total of 963 patients with Siewert-type II/III AEG were included, of which 132 cases were in the laparoscopic gastrectomy group, and 831 cases were in the open gastrectomy group. After regrouping with PSM, 132 patients in the laparoscopic gastrectomy group were balanced with 264 similar patients in the open gastrectomy group. As expected, the laparoscopic gastrectomy group had significantly longer operation times, but less blood loss. Furthermore, the two groups showed similar results for post-operative complications, duration of hospital stay and 2-year OS rate. Combined organ resection was an independent risk factor for 2-year OS rate.

**Conclusion:**

This study suggests that laparoscopic gastrectomy may serve as a safe and feasible treatment for Siewert-type II/III AEG and achieve similar oncologic outcomes as open gastrectomy.

## Introduction

Gastric cancer is the fourth most common malignant tumor in the world [1]. According to previous research, Siewert-type II/III AEG spreads rapidly, has a bad prognosis, and is mainly treated as proximal gastric cancer [2–3]. Surgical resection with a D2 lymphadenectomy is the major form of treatment for proximal gastric cancer [4]. Open gastrectomy plays a pivotal role in treating both early and advanced gastric cancers [5]. With the development of minimally invasive surgery, the number of laparoscopic gastrectomy procedures has increased in recent years [6]. However, whether laparoscopic gastrectomy should be the standard method for treating Siewert-type II/III AEG is still controversial.

There are several technical difficulties involved with laparoscopic gastrectomy for AEG, including sufficient lymph node dissection, complex structures of vasculature, and combined resection of other organs. Due to these difficulties, only a limited number of surgeons choose to perform laparoscopic gastrectomy. However, some researchers have reported that laparoscopic gastrectomy resulted in better short-term outcomes for treating early-stage Siewert-type II/III AEG [7]. Further research is needed to clarify the safety and feasibility of using laparoscopic surgery to treat advanced AEG, as well as the long-term outcomes for both early and advanced AEG. In this study, we aimed to examine the short- and long-term effects of using laparoscopic surgery to treat patients with Siewert-type II/III AEG, compared to the open surgery method using PSM.

## Patients and methods

### Ethics statement

The study was approved by ethics committee of Shanxi Cancer Hospital before it began. All participants signed a written informed consent. All data has been anonymized and de-identified.

### Patients

From January 2013 to December 2015, we retrospectively reviewed patients diagnosed with Siewert-type II/III AEG who were treated with open gastrectomy or laparoscopic gastrectomy at the Shanxi Cancer Hospital in China. The inclusion criteria were as follows: 1) histologically confirmed Siewert-type II/III AEG; 2) open or laparoscopic approaches with total or proximal gastrectomy; 3) primary R0 resection; 4) esophageal invasion < 3cm; 5) tumor size < 4cm; 6) transabdominal approaches; 7) no distal metastasis; 8) complete and accessible medical records. The exclusion criteria included: 1) multiple malignancies; 2) post-operative residual cancer (R1/R2 resection); 3) endoscopic resection for early gastric cancer; 4) use of transthoracic surgical approaches; 5) loss of follow-up data within two years. A total of 963 patients were enrolled in the study (laparoscopic group = 132, open group = 831). There is no established guideline to state an absolute indication regarding laparoscopic approach for Siewert-type II/III AEG. A widely accepted indication for laparoscopic approach was related to tumor size and surgical history, to achieve R0 resection. The attending surgeon determined the laparoscopic approach mainly depending on a history without surgery because of a fixed tumor size for two groups. Meanwhile, the possible complications as well as advantages and disadvantages of the 2 gastrectomy methods were informed to patients and their families. All patients received a gastrectomy with D2 lymphadenectomy in accordance with the Japanese gastric cancer guidelines [5]. Six to eight cycles of adjuvant chemotherapy were administered to those patients who were in an advanced stage beyond the T2 stage or who had lymph-node metastasis in any T stage. After the surgery, patient follow-up was every 3 months for the following three years.

Recorded clinical characteristics of the patients included: age, gender, body mass index, smoking/drinking history, past medical history, ECOG score, UICC stage, administration of neoadjuvant chemotherapy, surgical approach, duration of operation, number of lymph nodes harvested, combined resection of other organs, the type of gastrointestinal tract reconstruction (Roux-en-Y, Esophagogastrostomy, jejunum interposition / antrum-preserving single tract reconstruction), amount of blood lost during the operation, post-operation complications (anastomosis leakage, empyema, Intra-abdominal abscess, pneumonia), and duration of hospitalization.

### Data extraction and statistics

Propensity score matching analyses were used to minimize intergroup disparities and control for selection bias. Multivariable logistic regression was performed on both laparoscopic and open group using all variables with possible influence on the patients’ survival. Variables included in the multivariable logistic regression include gender, age, height, bodyweight, body mass index, smoking/drinking history, cardiovascular disease, diabetes, ECOG score, UICC stage, operation time, loss of blood, lymph node dissection number, combined organ resection, reconstruction type, post-operation complications, and hospital stay. A propensity score was then estimated for all subjects using this logistic regression, and open group patients were matched to laparoscopic group patients using the nearest neighbor matching within a caliper of 0.15 times the standard deviation of the propensity score. Prognostic factors and survival were estimated by the uni-and multivariable Cox proportional hazards model. The multivariable model was selected using the backward variable elimination technique with an elimination criterion of p value <0.05.

## Results

### Clinico-pathological characteristics of patients

A total of 132 patients were enrolled in the laparoscopic group, and 831 patients were enrolled in the open group. Baseline characteristics and clinical features of the participants are presented in Table 1. The two groups were comparable in terms of age, gender, body mass index, smoking/drinking history, past medical history, ECOG score, UICC stage, administration of neoadjuvant chemotherapy, number of lymph-nodes harvested, combined resection of other organs, blood lost during the operation, duration of hospitalization, and post-operation complications, including anastomosis leakage, empyema, and intra-abdominal abscess. Before PSM, the laparoscopic group had significantly longer operation times, a lower proportion of post-operation pneumonia, and different reconstruction types. Therefore, these factors were used as matching factors. The PSM technique matched 132 patients in the laparoscopic group to 264 open gastrectomy patients. After PSM, the laparoscopic group showed significantly longer operation times, less blood loss during surgery and a lower frequency of combined organ resection.

**Table 1:** Clinicopathological characteristics for patients in two cohorts before and after propensity score matching.

	Before propensity score matching			After propensity score matching		
	Laparoscopic group N = 132	Open group N = 831	*P*	Laparoscopic group N = 132	Open group N = 264	*P*
**Gender**			0.648			1.000
Male	114(86.4%)	705(84.8%)		114(86.4%)	228(86.4%)	
female	18(13.6%)	126(15.2%)		18(13.6%)	36(13.6%)	
**Age**	61(37,79)	62(28,87)	0.083	60.08±8.37	60.54±9.06	0.588
**Height**	167(150,182)	167(144,188)	0.912	167(150,182)	168(147,182)	0.300
**Body weight**	63(44,94)	63(38,102)	0.716	63(44,94)	65(38,97)	0.371
**Body Mass Index**	23(17.19,33.78)	22.95(14.68,35.36)	0.915	23.13±3.09	23.29±3.27	0.463
**Loss of body weight**			0.842			0.489
<10%	120(90.9%)	750(90.4%)		120(90.9%)	234(88.6%)	
≧10%	12(9.1%)	80(9.6%)		12(9.1%)	30(11.4%)	
**Smoking history**	68(51.5%)	387(46.6%)	0.290	68(51.5%)	112(42.4%)	0.087
**Drinking history**	33(25.0%)	181(21.8%)	0.409	33(25.0%)	58(22.0%)	0.499
**Cardiovascular Disease**	2(1.5%)	32(3.9%)	0.273	2(1.5%)	15(5.7%)	0.054
**Diabetes**	10(7.6%)	72(8.7%)	0.677	10(7.6%)	21(8.0%)	0.895
**ECOG score**			0.129			0.438
≦2	119(90.2%)	708(85.2%)		119(90.2%)	231(87.5%)	
>2	13(9.8%)	123(14.8%)		13(9.8%)	33(12.5%)	
**Neo-adjuvant chemotherapy**	3(2.3%)	16(1.9%)	1.000	3(2.3%)	6(2.3%)	1.000
**UICC stage**			0.614			0.125
**I**	28(21.2%)	93(11.2%)		28(21.2%)	40(15.2%)	
**II**	50(37.7%)	309(37.2%)		50(37.7%)	103(39.0%)	
**III**	54(40.9%)	429(51.6%)		54(40.9%)	121(45.8%)	
**Operation time (min)**	210(80,360)	160(60,460)	0.000	210(80,360)	180(120,460)	0.001
**Loss of blood (ml)**	100(20,1000)	100(10,3000)	0.185	100(20,1000)	150(10,3000)	0.018
**lymph node dissection number**	19(3,54)	19(1,67)	0.485	19(3,54)	20(2,62)	0.055
**Combined organ resection**	11(8.3%)	105(12.6%)	0.158	11(8.3%)	50(18.9%)	0.006
**Reconstruction type**			0.000			1.000
Roux-en-Y	112(84.8%)	536(64.6%)		112(84.8%)	223(84.8%)	
Esophagogastrostomy	20(15.2%)	269(32.4%)		20(15.2%)	40(15.2%)	
Jejunum interposition	0	25(3.0%)		0	0	
**Post-operation complications**						
**Anastomotic leakage**	5(3.8%)	31(3.7%)	1.000	5(3.8%)	12(4.5%)	0.726
**Empyema**	2(1.5%)	8(1.0%)	0.905	2(1.5%)	4(1.5%)	1.000
**Abdominal abscess**	4(3.0%)	25(3.0%)	1.000	4(3.0%)	8(3.0%)	1.000
**Pneumonia**	2(1.5%)	49(5.9%)	0.037	2(1.5%)	6(2.3%)	0.900
**Hospital stay**	24(13,84)	24(8,233)	0.716	24(13,84)	26(15,132)	0.109
**OS rate (2 years)**	118(89%)	723(87%)	0.532	118(89%)	225(85%)	0.249

### Surgical approaches and post-operation complications

The laparoscopic group required a significantly longer surgery duration compared with open gastrectomy, despite using PSM. However, there was a tendency for patients in the open group to lose a larger amount of blood during the operations. Patients having jejunum interposition/antrum-preserving single tract reconstruction were administered only with open gastrectomy. Additionally, combined resection of other organs was more prevalent in the open group. After PSM, there were no significant differences between the two groups for post-operation complications and duration of hospital stay.

### Overall survival (OS) rate and prognostic factors

**Fig 1:**
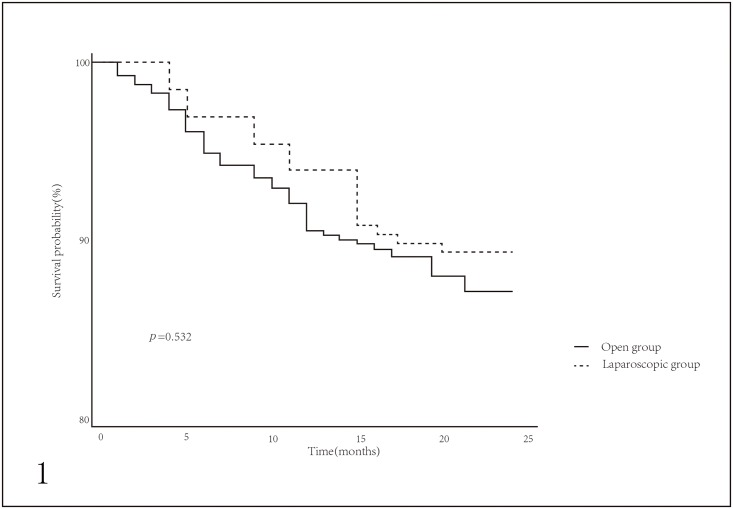
Two-year overall survival (OS) probability of patients in open group and laparoscopic group before propensity score matching.

**Fig 2:**
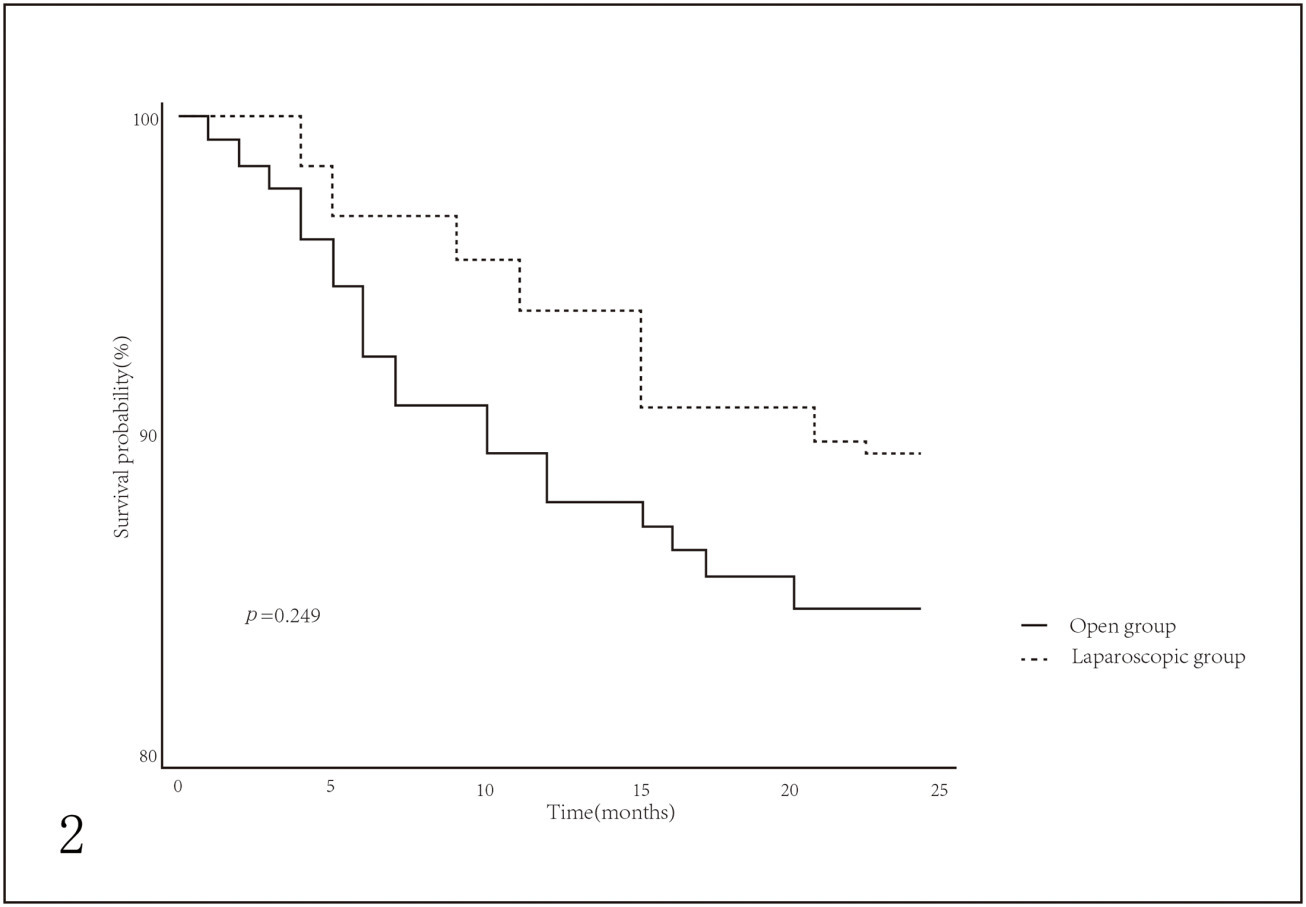
Two-year overall survival (OS) probability of patients in open group and laparoscopic group after propensity score matching.

The follow-up ranges from 4 to 49 months. The two-year overall survival (OS) were 89% in 118 laparoscopic patients, 87% in 732 unmatched open patients and 85% in 225 matched open patients. However, there were no significant differences for two-year OS between the two groups, neither in the unmatched cohort nor the matched cohort (Figs 1 and 2). Cox regression model revealed the results of the univariate and multivariate analyses. In unmatched cohort, post-operation pneumonia, UICC stage and combined organ resection were significantly associated with two-year OS in univariate analysis, while post-operation pneumonia and combined organ resection were independent predictors for two-year OS in multivariate analysis (Table 2). In matched cohort, UICC stage and combined organ resection were significantly associated with OS in univariate analyses after PSM, whereas combined organ resection was the only independent predictor for OS in multivariate analysis (Table 3).

**Table 2:** Cox regression analyses for unmatched patients: Uni-variable and multi-variable analysis.

	Uni-variable				Multi-variable			
	HR	95% CI		*P*	HR	95% CI		*P*
Gender	0.439	0.159	1.216	0.113				
Age	0.975	0.945	1.005	0.102				
Height	1.017	0.978	1.057	0.406				
Body weight	1.000	0.976	1.024	0.984				
Body Mass Index	0.984	0.908	1.067	0.696				
Loss of body weight≧10%	1.172	0.502	2.736	0.714				
Smoking history	1.001	0.589	1.699	0.998				
Drinking history	0.590	0.279	1.248	0.167				
Cardiovascular Disease	1.571	0.491	5.029	0.447				
Diabetes	0.617	0.193	1.975	0.416				
ECOG score	1.043	0.493	2.208	0.912				
Neo-adjuvant chemotherapy	1.957	0.477	8.030	0.351				
Surgical approach								
Laparoscopic group	1			0.534				0.702
Open group	0.764	0.327	1.783		0.847	0.361	1.986	
UICC stage								
I	1			0.032				
II	2.44	1.76	3.59	0.004				
III	3.69	2.13	5.37	0.045				
Operation time (min)	1.003	0.998	1.008	0.235				
Loss of blood (ml)	1.001	1.000	1.002	0.165				
lymph node dissection number	1.024	0.997	1.052	0.077				
Combined organ resection	2.300	1.235	4.284	0.009	2.258	1.210	4.212	0.010
Reconstruction type								
Roux-en-Y	1			0.213				
Esophagogastrostomy	0.553	0.285	1.070	0.079				
Jejunum interposition	0.000	0.000	2.301E+284	0.971				
Post-operation complications								
Anastomotic leakage	0.980	0.239	4.021	0.978				
Empyema	1.865	0.258	13.484	0.537				
Abdominal abscess	1.223	0.298	5.019	0.780				
Pneumonia	2.356	1.009	5.501	0.010	2.273	1.171	5.325	0.039
Hospital stay	1.006	0.991	1.021	0.460				

**Table 3:** Cox regression analyses for matched patients: Uni-variable and multi-variable analysis.

	Univariable				Multivariable			
	HR	95% CI		*P*	HR	95% CI		*P*
**Gender**	0.815	0.245	2.714	0.739				
**Age**	0.983	0.942	1.026	0.443				
**Height**	0.988	0.934	1.044	0.659				
**Body weight**	0.995	0.960	1.031	0.767				
**Body Mass Index**	0.996	0.884	1.122	0.945				
**Loss of body weight**≧10%	0.689	0.163	2.917	0.613				
**Smoking history**	1.034	0.478	2.235	0.933				
**Drinking history**	0.433	0.130	1.442	0.173				
**Cardiovascular Disease**	1.884	0.445	7.974	0.389				
**Diabetes**	0.979	0.231	4.144	0.977				
**ECOG score**	1.410	0.486	4.091	0.528				
**Neo-adjuvant chemotherapy**	1.777	0.241	13.114	0.573				
**Surgical approach**								
**Laparoscopic group**	1			0.255				0.390
**Open group**	0.589	0.236	1.466		0.666	0.264	1.681	
**UICC stage**								
**I**	1			0.069				
**II**	2.09	1.17	4.52	0.011				
**III**	4.01	1.97	6.12	0.027				
**Operation time (min)**	1.003	0.995	1.010	0.453				
**Loss of blood (ml)**	1.001	0.999	1.002	0.310				
**lymph node dissection number**	1.024	0.987	1.063	0.201				
**Combined organ resection**	2.461	1.070	5.661	0.024	2.293	0.985	5.339	0.046
**Reconstruction type**								
Roux-en-Y	1			0.378				
Esophagogastrostomy	0.038	0.000	3.768	0.164				
Jejunum interposition								
**Post-operation complications**								
**Anastomotic leakage**	1.943	0.459	8.220	0.367				
**Empyema**	2.900	0.393	21.402	0.297				
**Abdominal abscess**	2.806	0.663	11.875	0.161				
**Pneumonia**	0.048	0.000	7.125	0.621				
**Hospital stay**	1.016	0.995	1.038	0.137				

## Discussion

The prevalence of Siewert-type II/III AGE is increasing rapidly, and most patients are diagnosed at an advanced stage [3,8–9]. Previous retrospective studies and meta-analyses have revealed that patients receiving laparoscopic surgery show reduced blood loss, faster recovery, and fewer post-operative complications but have a significantly longer operation time compared to patents receiving open gastrectomy surgeries. Furthermore, the laparoscopic and open groups showed similar results in lymph node dissection and OS [10–12]. In our study, we also found that laparoscopic gastrectomy required a significantly longer operation time after PSM, which is consistent with previous studies [10–11]. This result is likely attributable to the technical difficulties of laparoscopic gastrectomy, such as sufficient lymph node dissection, complex vasculature structures, and combined resection of other organs. However, as experience grows with laparoscopic procedures, laparoscopic gastrectomy is no longer regarded as limited in its application, but rather has become a controversial approach [13].

According to the Japanese gastric cancer guidelines, total gastrectomy with D2 lymphadenectomy is the standard option for advanced gastric cancers [5]. It was reported that 60% to 70% of proximal gastric cancers are treated with chemotherapy after proximal gastrectomy with esophago-gastrostomy and antrum-preserving signal tract reconstruction [14]. In this study, some patients in both groups underwent proximal gastrectomy and esophagogastrostomy.

The results of our study suggest that blood loss was greater in the open group after PSM, which is probably attributable to the high ratio of combined organ resections and poor vessel exposure in open gastrectomy [15]. Our study showed no significant differences in the number of lymph nodes dissected before PSM or after PSM, which is consistent with previous studies. This result suggests that laparoscopic gastrectomy is safe and acceptable in the short-term [10–11,13,16].

Post-operation complications are also associated with surgical safety [12]. The results of a study by Mikito et al. suggest that laparoscopic gastrectomy is associated with a lower incidence of complications than open gastrectomy. We observed similar results in our study, though there was no significant difference between the two groups. However, we speculate that this may attributable to the fact that the type of surgery performed was chose by the patients and not their doctors.

The majority of recurrences or metastases occurred during the first two years after surgery [17]. There was no significant difference in the 2-year Overall survival (OS) rate between the two groups, which means the laparoscopic gastrectomy is safe and feasible for treating Siewert-type II/III AEG. Several retrospective studies and meta-analyses also reported that laparoscopic surgery was as safe as open surgery after a 5-year follow-up period [10–11,13].

PSM was initially proposed as a method for reducing bias in non-randomized control studies [18]. We aimed to reduce heterogeneity by incorporating all clinico-pathological factors into the PSM analysis. The independent prognostic factors for survival were: UICC stage, regardless of combined organ resection in both the unmatched and matched cohorts. The results may be explained by the fact that survival time was mainly determined by the UICCs [5]. On the other hand, a previous study has demonstrated that Siewert-type III AEG with tumor diameters >4 cm has a high relative frequency of splenic hilar metastasis [19]. Therefore, AEG often combined splenic hilar resection in this study. Additionally, metastases of the No. 10 lymph-node may be associated with a more advanced disease stage and a poor prognosis.

This study has some limitations. First, data were collected retrospectively, introducing some underlying selection bias into the study cohort. Second, surgical approaches were mainly decided by patients and not the doctors. More so, partial patients in both groups underwent proximal gastrectomy and esophagogastrostomy, which are not the best surgical approaches. Additionally, we did not achieve a 5-year follow-up time to evaluate the long-term survival of patients. Finally, other variables such as pathological and biological factors may introduce potential bias into the data.

In conclusion, the laparoscopic gastrectomy was a safe and feasible treatment for Siewert-type II/III AEG. Further prospective studies are necessary to confirm our results.
